# Proximal tubule reabsorptive dysfunction and risk of cardiovascular death among community-living adults: the HUNT-3 cohort

**DOI:** 10.1093/ehjopen/oeag091

**Published:** 2026-06-02

**Authors:** Jesse C Ikeme, Ronit Katz, Marius A Øvrehus, Knut Asbjørn R Langlo, Michelle M Estrella, Michael G Shlipak, Joachim H Ix, Stein I Hallan

**Affiliations:** Kidney Health Research Collaborative, University of California San Francisco and San Francisco Veterans Affairs Healthcare System, 4150 Clement St, Building 2, Room 140, San Francisco, CA 94121, USA; Department of Obstetrics & Gynecology, University of Washington, Seattle, WA, USA; Department of Clinical and Molecular Medicine, Faculty of Medicine and Health Sciences, Norwegian University of Science and Technology, Postboks 8900, Torgarden, NO-7491 Trondheim, Norway; Department of Nephrology, St. Olavs Hospital, Postbox 3250, Torgarden, NO-7006 Trondheim, Norway; Department of Clinical and Molecular Medicine, Faculty of Medicine and Health Sciences, Norwegian University of Science and Technology, Postboks 8900, Torgarden, NO-7491 Trondheim, Norway; Department of Nephrology, St. Olavs Hospital, Postbox 3250, Torgarden, NO-7006 Trondheim, Norway; Kidney Health Research Collaborative, University of California San Francisco and San Francisco Veterans Affairs Healthcare System, 4150 Clement St, Building 2, Room 140, San Francisco, CA 94121, USA; Kidney Health Research Collaborative, University of California San Francisco and San Francisco Veterans Affairs Healthcare System, 4150 Clement St, Building 2, Room 140, San Francisco, CA 94121, USA; Division of Nephrology-Hypertension, Department of Medicine, University of California San Diego; and Veterans Affairs San Diego Healthcare System, 200 W. Arbor Dr, San Diego, CA 92103, USA; Department of Clinical and Molecular Medicine, Faculty of Medicine and Health Sciences, Norwegian University of Science and Technology, Postboks 8900, Torgarden, NO-7491 Trondheim, Norway; Department of Nephrology, St. Olavs Hospital, Postbox 3250, Torgarden, NO-7006 Trondheim, Norway

**Keywords:** Alpha-1-microglobulin, Cardiovascular disease, Proximal kidney tubules, Biomarkers, Mortality

## Abstract

**Aims:**

Proximal tubule dysfunction in the kidney is associated with cardiovascular disease (CVD) in populations with prevalent chronic kidney disease (CKD). We sought to evaluate the nature of this association in the general population.

**Methods and results:**

We conducted a case-cohort study within the North Trøndelag Health Study (HUNT-3). We randomly sampled 1246 participants and all cases of cardiovascular death. Alpha-1 microglobulin (A1M) and beta-2 microglobulin (B2M) were measured in urine to assess proximal tubule reabsorptive dysfunction. Cardiovascular death was assessed using the Norwegian Cause of Death registry. We estimated the association of each biomarker with cardiovascular death after adjustment for baseline characteristics, including estimated glomerular filtration rate (eGFR) and albuminuria. Mean participant age was 51 years, 55% were female. Median urine albumin-to-creatinine ratio was 1.3 [interquartile range (IQR) 1.0–1.8] mg/mmol and mean eGFR was 98 (SD 17) mL/min/1.73 m^2^. Cardiovascular death occurred in 166 participants over a median follow-up of 13 years. The weighted incidence among those with low-normal, mid-normal, high-normal, and elevated A1M was 0.8 (0.6–1.1), 1.5 (1.0–2.0), 2.0 (1.3–2.6), and 4.5 (2.6–6.4) per cent, respectively. After adjustment for baseline characteristics, 1-SD higher urine A1M [hazard ratio (HR) 1.33, 95% confidence interval (CI) 1.12–1.58] and urine B2M (HR 1.28, 95% CI 1.10–1.48) were each associated with higher risk of cardiovascular death. We did not observe interaction across subgroups based on age, sex, albuminuria, or prevalent CVD.

**Conclusion:**

In a population-based cohort of adults, proximal tubule reabsorptive dysfunction, as indicated by elevated levels of urinary A1M and B2M, was associated with higher risk of cardiovascular death independent of eGFR and albuminuria.

## Introduction

Chronic kidney disease (CKD) is a well-established risk factor for cardiovascular disease (CVD).^1^ Lower levels of estimated glomerular filtration rate (eGFR) and higher levels of albuminuria are each independently associated with higher CVD risk; however, they only reflect the capacity and integrity of the glomeruli and fail to account for other components of kidney health.^2^

Tubule function is responsible for active solute and water handling by the kidney, making it a critical component of kidney health. Specifically, dysfunctional proximal tubule handling of low molecular weight proteins, as observed in persons exposed to tubule-toxic medications or heavy metals,^3–5^ is thought to contribute to tubulointerstitial inflammation, fibrosis, and CKD progression.^6–10^ By measuring urinary levels of low molecular weight proteins that are typically reabsorbed in the proximal tubule, including alpha-1-microglobulin (A1M) and beta-2-microglobulin (B2M), proximal tubule health may be assessed, providing an additional dimension of kidney health which could contribute to a more comprehensive assessment of kidney disease and its entailing risks.

Proximal tubule reabsorptive dysfunction has previously been associated with CVD, but existing evidence comes almost exclusively from cohorts selected for prevalent kidney or CVD.^8,11–17^ We recently demonstrated that impaired proximal tubule dysfunction measured in the general population is associated with major adverse kidney events independent of CKD risk factors, eGFR, and albuminuria.^18^ The association of proximal tubule reabsorptive dysfunction among the general population with CVD risk is largely unknown. Therefore, we evaluated urine biomarkers A1M and B2M and their association with risk for cardiovascular death within a population-based cohort in Norway.

## Methods

### Study design

We conducted a case-cohort analysis within The Trøndelag Health Study (HUNT). HUNT is a population-based study consisting of decennial demographic and clinical assessments paired with urine and blood collection among all adults in North Trøndelag county of Norway. The study began in 1986, with the third iteration inviting adults between 2006 and 2008. It has benefited from a high rate of participation among eligible adults.^19^ Data from the study have been linked to electronic health records and several health and administrative data registries. The current study was approved by the Regional Committee for Medical Research Ethics.

### Study population

Out of 93 860 eligible residents of North Trøndelag County who were invited, 50 807 (54.1%) participated in HUNT-3. Complete blood and urine samples were available from 11 878 participants. From those, we selected a random 10% sample as the subcohort for our study. We additionally selected all participants with samples available who experienced cardiovascular death during follow-up as case subjects.

### Proximal tubule biomarkers

Mid-stream urine samples were collected from participants at their study examination, frozen within 15 min, then stored at −80°C within 2 h. Alpha-1 microglobulin and beta-2 microglobulin were measured at Levanger Hospital and HUNT biobank, respectively. A1M was measured using a BNII nephelometer (Siemens, Germany), with a lower limit of detection of 5.6 mg/L and a coefficient of variation of 1.8% at 38 mg/L and 6.7% 15 mg/L. B2M was measured on a MESO QuickPlex SQ 120 multiplex system (Meso Scale Discovery, USA), with a coefficient of variation of 6.9%.

### Other characteristics

Medical history was assessed via questionnaire. History of diabetes was defined as having a diagnosis made by a physician, use of an antidiabetic medication, or having a non-fasting serum glucose above 11 mmol/L (199 mg/dL) at the time of the HUNT-3 assessment. Use of antihypertensive medications was recorded. History of CVD included prior angina pectoris, percutaneous coronary intervention or coronary bypass operation, myocardial infarction, heart failure, haemorrhagic or ischaemic stroke, transient ischaemic attack, or central or peripheral arterial disease. Smoking status was reported as never, former, or current. Body mass index (BMI) was calculated from height and weight. Blood pressure is reported as the average of three consecutive blood pressure measurements obtained during the study visit. Total cholesterol, high-density lipoprotein, triglycerides, and creatinine were measured in a blood sample collected during the study visit. Urine albumin-to-creatinine ratio was calculated. Estimated GFR was determined using the creatinine-based 2009 CKD-EPI equation, the equation currently used in Norway.

### Outcomes

HUNT-3 data were linked with the Norwegian Cause of Death Registry. End of observation was 1 September 2020. Cardiovascular death was determined based on the primary cause of death being reported as a CVD-related diagnosis (ICD-10 codes I10-I79; i.e, hypertensive, ischaemic, pulmonary, and other heart diseases; cerebrovascular diseases; and diseases of arteries). The Norwegian Cause of Death registry captures over 98% of deaths in the country and ranks among the best in the world.^20,21^ The accuracy of multiple CVD diagnoses obtained from national registries of the Norwegian healthcare system has been validated as accurate and complete against outcomes adjudicated in prospective cohorts.^22,23^

### Statistical analysis

We summarized baseline characteristics using means and standard deviations (SD). Because urine albumin is typically right-skewed, we reported the median and interquartile range. For each biomarker, we defined the normal range as below the 97.5th percentile among healthy subcohort participants, which has been defined in HUNT as age 18–80 years, reporting good or excellent general health and without diabetes, CVD, antihypertensive medication use, or current smoking, and with measured BMI <35 kg/m^2^, BP <140/90, eGFR >60 mL/min/1.73m^2^, and urine albumin-to-creatinine ratio (UACR) <3 mg/mmol (30 mg/g). ‘Low-normal’, ‘mid-normal’, and ‘high-normal’ categories were divided by the 60th and 80th percentiles of healthy participants in part because 58% of A1M measurements were below the measurable limit.

Cox proportional hazards regression was used to estimate associations between each biomarker and cardiovascular death. Analyses accounted for the case–cohort design by applying sampling weights equal to the inverse of each participant’s sampling fraction: Participants in the random subcohort were assigned a weight of 10, and additional case subjects were assigned a weight of 0 before failure, and 1 at the time of cardiovascular death. To assess the functional form of these associations, we modelled the hazard ratio for CVD death adjusted for urine creatinine using restricted cubic splines. We estimated the association between a 1-SD higher concentration in each proximal tubule biomarker with cardiovascular death. We also estimated the association between the percentile-based categories of each biomarker with cardiovascular death, using the low-normal category as a reference. Regression models were sequentially adjusted: first for urine creatinine; then for age, sex, systolic blood pressure, antihypertensive use, BMI, smoking, cholesterol, diabetes, and prevalent CVD; and finally, for eGFR and urine albumin. Similar regression models were then performed with testing for interaction across subgroups of interest based on age, sex, prevalent hypertension, prevalent CVD, UACR, and eGFR categories in exploratory analyses. All statistical analyses were performed using Stata version 17 (StataCorp LLC, College Station, TX, USA).

## Results

### Baseline characteristics

**Figure 1:**
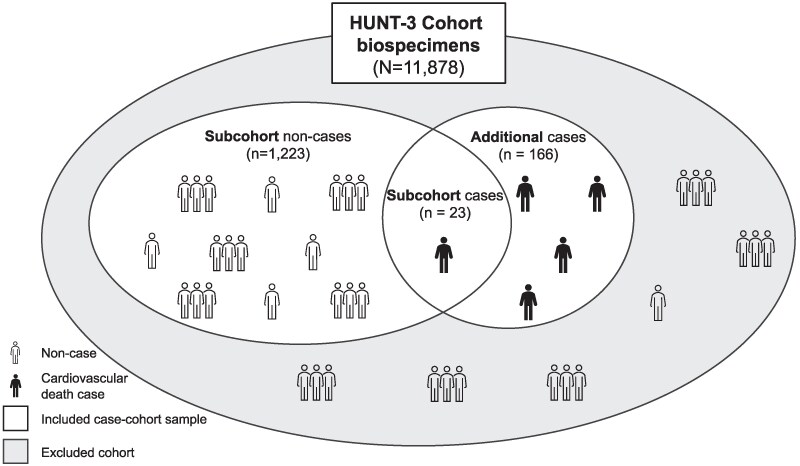
Study design and patient inclusion.

Among the 1246 participants in the subcohort (*Figure 1*), the mean age was 51 (SD 14) years and 46% were male. When surveyed, 3.7% had diabetes, 6.2% had prevalent CVD, and 16% were using anti-hypertensive medications. Median UACR was 1.3 mg/mmol [interquartile range (IQR) 1.0–1.6] and mean eGFR was 98 (SD 17) mL/min/1.73 m^2^ (*Table 1*). Baseline characteristics within the subcohort were generally similar to the total HUNT cohort (see Supplementary material online, *Table S1*). Median (IQR) of A1M and B2M in the subcohort were 5.6 (5.6–10.0) mg/L and 0.47 (0.22–0.79) mg/L, respectively. Biomarker categories are summarized in *Table 2*. Spearman correlation between urine A1M and B2M was 0.55 with weaker correlation observed between either biomarker and eGFR or UACR (see Supplementary material online, *Table S2*).

**Table 1:** Baseline characteristics of random HUNT-3 subcohort and additional included cases

	Random subcohort (*n* = 1246)		Additional cases (*n* = 166)	
Characteristic	*n* or mean	(SD or %)	*n* or mean	(SD or %)
Age (years)	51.0	(13.9)	70.1	(11.2)
Male	567	(45.5%)	98	(59.0%)
Diabetes mellitus	46	(3.7%)	18	(10.8%)
Diabetes duration, years	8.4	(7.1)	9.5	(7.5)
Cardiovascular disease	77	(6.2%)	40	(24.1%)
Angina Pectoris	28	(2.2%)	14	(8.4%)
PCI/CABG	31	(2.5%)	18	(10.8%)
Myocardial Infarction	35	(2.8%)	23	(13.9%)
Heart Failure	12	(1.0%)	14	(8.4%)
Other heart disease	42	(3.4%)	19	(11.4%)
Stroke	22	(1.8%)	10	(6.0%)
Systolic blood pressure, mmHg	130.6	(17.1)	140.4	(20.1)
Antihypertensive use	201	(16.1%)	85	(51.2%)
Smoking				
Never	515	(41.3%)	63	(38.0%)
Former	354	(28.4%)	49	(29.5%)
Current	249	(20.0%)	39	(23.5%)
Missing	128	(10.3%)	15	(9.0%)
BMI, kg/m^2^	26.8	(4.2)	27.1	(4.0)
Total cholesterol, mmol/L	5.5	(1.1)	5.5	(1.3)
HDL cholesterol, mmol/L	1.4	(0.4)	1.3	(0.4)
Triglycerides, mmol/L	1.6	(1.0)	1.8	(1.0)
UACR, mg/mmol	1.3	(1.0–1.6)	1.9	(1.3–2.6)
eGFR, mL/min/1.73 m^2^	98.4	(16.8)	80.1	(19.1)

UACR reported as median and IQR.

PCI, percutaneous coronary intervention; CABG, coronary artery bypass graft; BMI, body-mass index; HDL, high-density lipoprotein; IQR, interquartile range; UACR, urine albumin-to-creatinine ratio; GFR, glomerular filtration rate.

**Table 2:** Cardiovascular death by level of urinary alpha-1-microglobulin and beta-2-microglobulin among HUNT-3 participants

Urinary biomarker	Category	Range, mg/L	Events/*n* (subcohort)	Additional cases	Weighted incidence, % (95% CI)
A1M	Low-normal	<5.6	10/680	46	0.8 (0.6–1.1)
	Mid-normal	5.6–9.9	5/258	31	1.5 (1.0–2.0)
	High-normal	10.0–22.1	4/240	41	2.0 (1.3–2.6)
	Elevated	>22.1	4/68	25	4.5 (2.6–6.4)
B2M	Low-normal	<0.6	10/763	87	1.3 (1.0–1.6)
	Mid-normal	0.6–0.9	7/234	17	1.1 (0.6–1.5)
	High-normal	1.0–2.1	3/212	27	1.4 (0.9–2.0)
	Elevated	>2.1	3/37	12	4.4 (1.8–7.0)

Low-normal, mid-normal, high-normal, and elevated categories correspond to percentiles <60, 60–80, 80–97.5, and >97.5 among healthy participants.

A1M, alpha-1-microglobulin; B2M, beta-2-microglobulin.

### Urine tubule biomarkers and cardiovascular death

There were 166 cases of cardiovascular death during follow-up over a median of 12.9 years among the 11 878 individuals who provided blood and urine samples. The cardiovascular deaths included 23 participants who were randomly sampled in the subcohort (1.8% of subcohort), with the remaining 143 occurring among participants not in the subcohort. Persons with the highest levels of urine A1M and B2M had the highest incidence of cardiovascular death (*Table 2*). These associations appeared to extend through the detectable range (see Supplementary material online, *Figure S1*).

In separate Cox proportional hazards regression models, 1-SD higher urine A1M [hazard ratio (HR) 1.33, 95% confidence interval (CI) 1.12–1.58] and urine B2M (HR 1.28, 95% CI 1.10–1.48) were associated with higher risk of cardiovascular death after adjustment for baseline clinical and demographic characteristics, including eGFR and urine albumin (*Table 3*).

**Table 3:** Association of proximal tubular reabsorption biomarkers with cardiovascular death in the general population

Biomarker	Category	Model 1			Model 2			Model 3		
		HR	95% CI	*P*	HR	95% CI	*P*	HR	95% CI	*P*
A1M	1 SD	**1.32**	**(1.13–1.55)**	**0**.**001**	**1**.**32**	**(1.13–1.55)**	**0**.**001**	**1**.**33**	**(1.12–1.58)**	**0**.**001**
	Low-normal	1.00	Ref.		1.00	Ref.		1.00	Ref.	
	Mid-normal	1.20	(0.72–2.03)	0.48	1.42	(0.80–2.51)	0.23	1.42	(0.80–2.52)	0.23
	High-normal	1.08	(0.62–1.89)	0.78	1.28	(0.70–2.33)	0.42	1.26	(0.69–2.31)	0.45
	Elevated	1.69	(0.78–3.66)	0.19	**2**.**31**	**(1.06–5.01)**	**0**.**04**	**2**.**29**	**(1.05–5.03)**	**0**.**04**
										
B2M	1 SD	**1**.**20**	**(1.04–1.39)**	**0**.**013**	**1**.**27**	**(1.11–1.45)**	**0**.**001**	**1**.**28**	**(1.10–1.48)**	**0**.**001**
	Low-normal	1.00	Ref.		1.00	Ref.		1.00	Ref.	
	Mid-normal	0.82	(0.46–1.46)	0.50	0.82	(0.43–1.56)	0.55	0.83	(0.43–1.58)	0.57
	High-normal	0.76	(0.44–1.31)	0.32	0.98	(0.57–1.68)	0.93	0.98	(0.57–1.68)	0.93
	Elevated	**2**.**66**	**(1.33–5.33)**	**0**.**006**	**3**.**82**	**(1.93–7.59)**	**<0**.**001**	**3**.**83**	**(1.91–7.68)**	**<0**.**001**

Low-normal, mid-normal, high-normal and elevated categories correspond to percentiles <60, 60–80, 80–97.5, and >97.5 among healthy participants. Model 1: adjusted for urine creatinine, age, and sex; Model 2: adjusted for model 1 plus systolic blood pressure, antihypertensive use, BMI, smoking, cholesterol, diabetes, and cardiovascular disease. Model 3: adjusted for model 2 plus eGFR and urine albumin. Values with *P* < 0.05 highlighted in bold.

A1M, alpha-1-microglobulin; B2M, beta-2-microglobulin.

When proximal tubule biomarker levels were categorized, those with urine A1M and B2M in the mid-normal and high-normal ranges did not have significantly higher risk of cardiovascular death than those in the low-normal range. However, those with abnormally elevated urine A1M and B2M levels, i.e. above the 97.5th percentile of healthy participants, had 2.29 (95% CI 1.05–5.03) and 3.83 (95% CI 1.91–7.68) times higher risk of cardiovascular death than those in the low-normal category, respectively (*Table 3*). When both markers were combined in the same adjusted model, the strengths of association for each marker were only modestly attenuated (HR 1.30, 95% CI 0.91–1.84 and HR 1.24, 95% CI 1.02–1.50 for A1M and B2M, respectively), and there was no significant interaction between them (*P* for interaction = 0.38).

**Figure 2:**
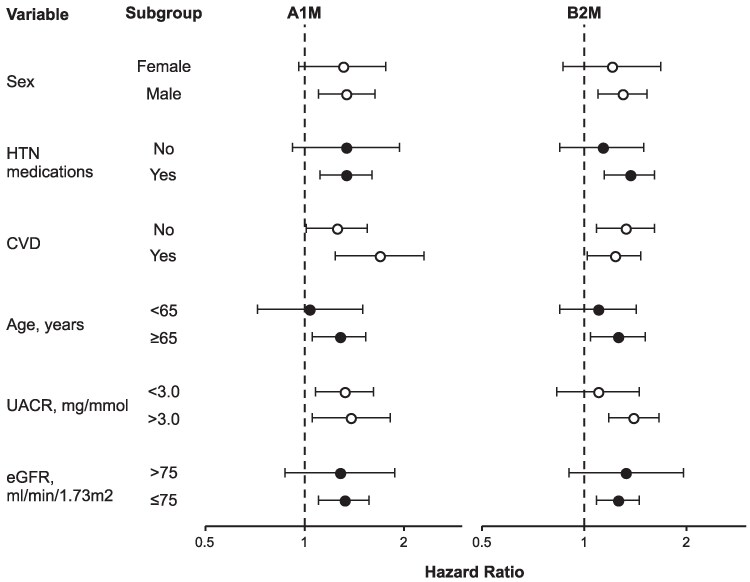
Association of alpha-1-microglobulin, beta-2-microglobulin and cardiovascular death across clinical subgroups. Hazard ratio and 95% confidence interval for 1-SD increase in biomarker depicted with adjustment for covariates and interaction across the variable of interest. A1M, alpha-1-microglobulin; B2M, beta-2-microglobulin; HTN, hypertension; CVD, cardiovascular disease; UACR, urine albumin-to-creatinine ratio; eGFR, estimated glomerular filtration rate.

Our analyses of A1M and B2M within demographic and clinical subgroups did not reveal significant interactions by age, sex, prevalent CVD, antihypertensive use, eGFR, or UACR (*Figure 2*).

## Discussion

In this analysis of markers of dysfunctional proximal tubule reabsorption and risk of CVD in a population-based cohort, we found that abnormally elevated levels of urinary A1M and B2M were associated with cardiovascular risk, even after adjustment for eGFR, albuminuria, and other risk factors. Associations were consistent across key subgroups, including those with and without prevalent CVD at baseline.

Greater urinary excretion of tubular reabsorption biomarkers has previously been found to associate with higher risk of CVD events in populations with prevalent CVD or CKD. Among hypertensive patients with CKD, both urinary A1M and B2M have been associated with CVD and all-cause mortality.^15,16^ Similar associations for urinary A1M have also been reported among persons with CKD in an area with endemic cadmium pollution,^24^ among kidney transplant recipients,^17^ and in elderly persons.^13^ However, very few papers have evaluated this important question in a relatively healthy, population-based cohort. Higher urine B2M was associated with cardiovascular death and all-cause mortality in a population-based cohort in Japan,^11^ but Framingham Heart Study participants did not demonstrate significant associations between higher urinary A1M or B2M and cardiovascular death.^25^ Both A1M and B2M are freely filtered in the glomerulus and reabsorbed in the proximal tubule; however, they are sufficiently different in terms of prior study, metabolic production, and stability in storage that their concordant association with CVD death allows a stronger inference about proximal tubule reabsorptive function. Our study supports the hypothesis that proximal tubule function, as measured by urine A1M and B2M, may be an additional dimension of kidney health that contributes to CVD risk in addition to eGFR and albuminuria. This also suggests that, in addition to detecting tubule injury from malignancies, medications, toxins, and urinary obstruction, proximal tubule reabsorptive biomarkers may be useful for cardiovascular risk assessment.^4,26–29^

The mechanisms underlying the association between proximal tubular reabsorptive dysfunction and cardiovascular mortality remain incompletely understood. Megalin, a critical endocytic receptor in the proximal tubule, is responsible for the reabsorption of A1M and B2M. It is also essential to the conservation of critical metabolic proteins, hormones, and vitamins, which may be necessary for optimal cardiovascular health.^30–33^ Proximal tubule reabsorptive dysfunction also results in proteinuria which may contribute to cardiovascular risk via downstream effects in the kidney such as tubulointerstitial inflammation and fibrosis. This is typically hypothesized to occur due to protein reabsorption; however, we have also previously reported an association between increased urinary A1M and B2M, elevated blood pressure, and worse renal outcomes, suggesting alternative mechanisms.^18,34,35^ Finally, proximal tubule dysfunction may be the result of otherwise unmeasured systemic processes leading to cardiovascular risk. While these mechanisms remain speculative, as the case is with GFR and albuminuria, the uncertainty regarding mechanism does not diminish the potential importance of this tubule axis of kidney health as a contributor to CVD beyond the accumulation of uremic toxins.^36^ Furthermore, these findings lend support to the hypothesis that the glomerular measures of eGFR and albuminuria provide an incomplete assessment of kidney.^2^

Our study has several strengths. The use of a population-based cohort with high participation and long follow-up extends previous findings of proximal tubule associated CVD risk from select high-risk populations to the wider general population. Our case–cohort design addresses the problem of low incidence in a general population-based study. Prompt freezing of specimens enabled the detection of an association involving B2M, which might otherwise degrade in urine samples with low pH. We adjusted for several variables, which we have previously reported are associated with urine A1M and B2M levels, including higher blood pressure, male sex, and antihypertensive use, though comorbidity assessment was largely by self-report and the possibility of residual confounding remains a relevant limitation.^35^ This study was also conducted in a Norwegian population, and no data were collected on participant ethnicity, which may limit generalizability to populations in other regions. Despite high participation, ascertainment bias is also a possibility; however, this would be expected to bias estimates toward the null.

In summary, proximal tubule reabsorptive dysfunction, as indicated by higher urine concentrations of A1M and B2M, is associated with cardiovascular death in the general population. The findings are independent of known CKD risk factors and glomerular markers of kidney health. These results contribute to the body of research evaluating novel means of assessing kidney health as part of more comprehensive cardiovascular risk assessment. Future studies are needed to confirm these findings in other population-based cohorts.

## Supplementary Material

oeag091_Supplementary_Data

## Data Availability

The data underlying this article will be shared on reasonable request to the corresponding author.
