# Supplementary material for: Proximal tubule reabsorptive dysfunction and risk of cardiovascular death among community-living adults: the HUNT-3 cohort
Source: Eur Heart J Open. 2026 Jun 2;6(3):oeag091. doi: 10.1093/ehjopen/oeag091 (PMC13262532; doi:10.1093/ehjopen/oeag091)
Supplement: oeag091_Supplementary_Data [file oeag091_supplementary_data.pdf]

Table S1: Baseline characteristics of HUNT-3 and sampled subcohort

Characteristic	Random subcohort (n = 1,246)		HUNT-3 biospecimens (n = 11,878)		HUNT-3 (N = 50,583)	
	N or mean	(SD or %)	N or mean	(SD or %)	N or mean	(SD or %)
Age, years	51.0	(13.9)	51.2	(13.8)	53.1	(16.1)
Male	567	(45.5%)	5,161	(43.5%)	22,929	(45.3%)
Diabetes Mellitus	46	(3.7%)	429	(3.6%)	2,354	(4.7%)
Blood glucose, mmol/L	5.5	(1.3)	5.5	(1.4)	5.6	(1.6)
CVD	72	(5.8%)	634	(5.3%)	3,883	(7.7%)
Systolic BP, mmHg	130.6	(17.1)	129.5	(16.9)	130.4	(18.7)
Diastolic BP, mmHg	74.5	(11.7)	73.9	(11.1)	73.2	(11.2)
Antihypertensive use	201	(16.1%)	1,957	(16.5%)	10,544	(20.8%)
Smoking						
Never	515	(41.3%)	5,019	(46.9%)	21,753	(48.0%)
Former	354	(28.4%)	3,479	(32.5%)	14,025	(31.0%)
Current	249	(20.0%)	2,200	(20.6%)	9,534	(21.0%)
BMI, kg/m^2	26.8	(4.2)	26.8	(4.2)	27.2	(4.4)
Total cholesterol, mmol/L	5.5	(1.1)	5.5	(1.1)	5.5	(1.1)
UACR, mg/mmol	1.3	(0.96-1.64)	1.5	(1.13-1.99)	1.3	(0.6 – 2.4)
eGFR, ml/min/1.73m^2	99	(89.6-109.5)	98.7	(88.6-108.7)	96.9	(84.8 – 107.9)

Abbreviations: BMI, body-mass index; UACR, urine albumin-to-creatinine ratio; eGFR, estimated glomerular filtration rate. UACR and eGFR reported as median and IQR.

Table S2: Spearman correlation of urinary proximal tubule biomarkers, eGFR, and UACR

	A1M	B2M	eGFR
B2M	0.545		
eGFR	-0.177	0.043	
UACR	0.184	0.013	-0.458

Abbreviations: A1m alpha-1-microglobulin, B2m, beta-2-microglobulin; UACR, urine albumin to creatinine ratio. Urine albumin, A1m, and B2m indexed to urine creatinine

Figure S1: Splines depicting the association of alpha-1-microglobulin, beta-2-microglobulin and cardiovascular death

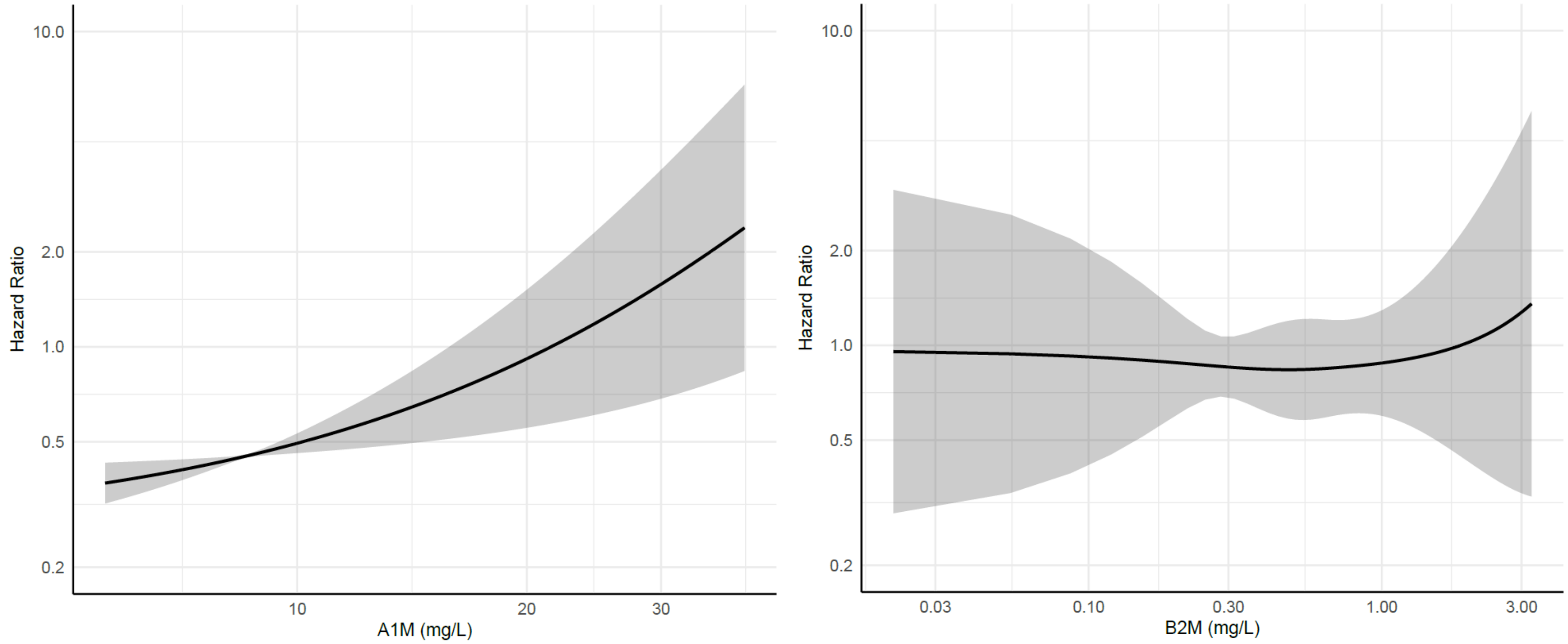

Abbreviations: A1M: alpha-1-microglobulin, B2M: beta-2-microglobulin. Plots depict A1M (left) and B2M (right) restricted cubic splines of hazard ratio for cardiovascular death adjusted for urine creatinine with knots at tertiles of B2M and the median A1M value among measurable samples. Shaded area represents 95% confidence interval.
